# *Taking Action Together*: A YMCA-based protocol to prevent Type-2 Diabetes in high-BMI inner-city African American children

**DOI:** 10.1186/1745-6215-11-60

**Published:** 2010-05-21

**Authors:** Lorrene D Ritchie, Sushma Sharma, Joanne P Ikeda, Rita A Mitchell, Aarthi Raman, Barbara S Green, Mark L Hudes, Sharon E Fleming

**Affiliations:** 1 Dr Robert C and Veronica Atkins Center for Weight and Health, University of California, Berkeley, CA 94720-3100, USA; 2 Department of Nutritional Sciences and Toxicology, University of California, Berkeley, CA 94720-3104, USA

## Abstract

**Background:**

Associated with a tripling in obesity since 1970, type 2 diabetes mellitus (T2DM) in children has risen 9-10 fold. There is a critical need of protocols for trials to prevent T2DM in children.

**Methods/Design:**

This protocol includes the theory, development, evaluation components and lessons learned from a novel YMCA-based T2DM prevention intervention designed specifically for high-BMI African American children from disadvantaged, inner-city neighborhoods of Oakland, California. The intervention was developed on the basis of: review of epidemiological and intervention studies of pediatric T2DM; a conceptual theory (social cognitive); a comprehensive examination of health promotion curricula designed for children; consultation with research, clinical experts and practitioners and; input from community partners. The intervention, *Taking Action Together*, included culturally sensitive and age-appropriate programming on: healthy eating; increasing physical activity and, improving self esteem.

**Discussion:**

Evaluations completed to date suggest that *Taking Action Together *may be an effective intervention, and results warrant an expanded evaluation effort. This protocol could be used in other community settings to reduce the risk of children developing T2DM and related health consequences.

**Trial registration:**

ClinicalTrials.gov NCT01039116.

## Background

There is a critical need for trials to be conducted that aim to identify strategies to prevent T2DM mellitus (T2DM) in children. Associated with a tripling in obesity since 1970, T2DM in children has risen 9-10 fold [1]. Additionally, nearly 1 in 6 overweight youth has pre-diabetes [2]. African American youth are among the highest for risk of T2DM with higher rates of obesity and insulin-resistance than other ethnic groups due, in part, to being more insulin resistant [3]. Nearly 50% of African American children born in the U.S. in 2000 are expected to develop diabetes in their lifetimes [4].

*Taking Action Together *(*TAT*) was a controlled community-based intervention protocol developed by researchers at U.C. Berkeley in partnership with the YMCA of the East Bay to reduce risk of T2DM among low-income, high BMI, 9-10 year old African American children through improvements in nutrient intake, physical activity, and self esteem. To avoid stigmatization due to body fatness, focus was placed on improving diet and physical activity rather than on weight loss. Described are the rationale, theory, design, development, implementation, evaluation components, and lessons learned from *TAT*.

This protocol was designed for delivery to low-income African American children living in disadvantaged, inner-city neighborhoods such as those of East or West Oakland, CA. Compared to a White child living in the Oakland hills (comparatively higher income area), those African American children are more likely to be born low birth weight, live in a low-income household, have parents with only a high school education or less, and have poor access to healthy foods [5].

There is a dearth of culturally sensitive interventions that target obesity, diabetes, diet or physical activity among African American youth [6], especially for those living in disadvantaged neighborhoods. Out-of-school settings represent a widely accessible, but largely untapped and under-researched, venue for T2DM prevention. In the U.S. there are ~3,000 YMCA sites serving nearly 10 million children [7]. Out-of-school programs typically have more flexibility than schools and may be better able to include health-related activities. While healthy benefits from out-of-school programs have been reported [8-12], more studies in 'real world' settings are needed to translate research findings into practice to stem the rapid increase in T2DM.

### Hypotheses and specific aims

Consistent with social cognitive theory, we postulated that development of self efficacy with respect to targeted behaviors would improve children's dietary intake, physical activity, and self esteem which, in turn, would reduce insulin resistance in part by stabilizing body weight. Thus, our main hypothesis was that children in the treatment group would show more favorable changes in insulin resistance than children in the control group following 1 or more 2 years in the program. A secondary hypotheses was that there would be greater improvements in the treatment group in dietary intakes and physical activity. A third hypothesis stated that early markers of behavioral change (dietary and physical activity self efficacy, self esteem, positive behaviors and communications) would be improved in the treatment group when compared to the control.

Thus, the specific aims were (1). To assess the influence of treatment group status on change in insulin resistance (fasting HOMA-IR), (2). To determine the influence of treatment group status on change in intermediate outcomes (diet and physical activity), and (3). To determine the influence of treatment group status on change in potential moderating or mediating variables (self esteem, self efficacy, psychobehavioral characteristics).

## Methods/Design

### Selection Criteria (eligibility)

Inclusion criteria for child participants included: ancestry including at least one African American biological parent; being 9 to 10 years old; having BMI at or above the 85th percentile [13]; free of any systematic disorder or medication known to affect energy metabolism or body weight; and free of severe physical or emotional conditions that could interfere with study participation. The lower limit placed on age was based on concerns that the parents of younger children may be reluctant to consent to a blood draw, and on our assessment that the ethical risk-benefit ratio was less favorable in younger children. The upper limit was placed to avoid having to sub-group children according to age, loosing statistical power. Broader age ranges would need to be evaluated in subsequent studies.

### Primary outcomes

Change in insulin resistance over 1 or 2 yr of intervention was the primary outcome measure. Fasting glucose and insulin values were used to calculate the homeostatic model parameter - HOMA-IR, defined as fasting glucose (mmol/l) × insulin (μU/ml)/22.5 [14] and used as an index of insulin resistance [15]. Fasting indices of insulin resistance have been shown to be well correlated with estimates obtained using the "gold standard" methods of assessing insulin resistance using the euglycemic-hyperinsulinemic clamp in 9 - 10 year old children [16] and oral glucose tolerance test in premenarchal girls [17], and these indices have been used to show that overweight children are at greater risk for type 2 diabetes than normal weight children [18]. Lower risks are associated with this fasting index since it requires only a single veinpuncture and, thus, is preferable for use in children. The study's second primary outcome, change in glycosylated hemoglobin (HbA1c), was assessed by quantifying HbA1c using a dual HPLC method which detects variants common in African Americans that interfere will interpretation of HbA1c data [19,20].

### Demographic and Secondary outcomes

*Anthropometric variables *(Table 1), including child body weight and height, were measured and used to calculate BMI and BMI z-score. Additionally, waist and hip circumferences, and percent body fat were measured.

**Table 1:** Evaluation Measures in *Taking Action Together*

Measurement	Procedure &/or reference
**Anthropometry (child)**	
Waist & hip circum and ratio	With plastic non-elastic tape [50]
Weight, height, BMI and BMIz	Wt: digital electronic scale, Ht: portable stadiometes, BMIz Calc [13]
% Body fat	BIA [50], % Body Fat Horlick's equation [51,52]
**Glucoregulation (Child)**	
Fasting Glucose, Insulin	Commercially available kits [50]
HOMA-IR	Calculation of insulin resistance [14]
HbA1c, C-Peptide, NEFA	HbA1c [19,20]; C-Pep & NEFA, (Linco & Wako kits).
Acanthosis nigricans	Appearance of skin on back of neck with 0-4 scale of severity [53]
**Diet (child)**	
Dietary intakes	Food diary [54,55]; average intake and servings [36]
Dietary habits & Nutrition knowledge	After-School Student Questionnaire self-efficacy [50,56]
**Physical Activity and Fitness (child)**	
Physical activity & fitness	3-day PA diaries [55], Lap run [57]
PA habits & knowledge	California Dept. of Education's Healthy Kids Survey [58]
Athletic competence	Harter self-perception profile for children [59]
**Self efficacy**	
Diet self-efficacy	ASSQ self-efficacy questions [56]
PA self-efficacy	Child's Self-Assessment for Physical Activity (CSAAPA) [57,60]
**Self-esteem & Body Image (child)**	
Self-worth & social acceptance	Harter self-perception profile for children [59]
Body satisfaction	Validated in African American adults [61], modified [62]
Self-esteem	Behavioral Assessment System for Children, BASC-2 [34], child report
**Behavior and Communications (child)**	
Conduct problems & activities	BASC-2 [34], parent report and child report
Communication & social skills	BASC-2 [34]; parent and child report
**Adult and Family Assessments**	
Family food behaviors	Shopping, meal preparation and family eating [63]
Change for diet and PA	Fruit & vegetables [64], avoidance of high-fat [64,65], exercise [66]
Family PA habits & weight	Family member's habits, duration of activity, and prevalence of overweight [50]
**Additional Potential Confounding Variables**	
Family environment & SEI	Moos Family Environment Scale [67], Socioeconomic index [63]
Family & Intrauterine history of T2DM	American Diabetes Association instrument [68]
Polyovarian syndrome	(female child participants only) [50]
Pubertal stage	Estradiol (E2), luteinizing hormone [50,69]

*Hematological variables *other than the primary outcomes previously described, included measurement of non-esterified fatty acid (NEFA) concentrations, which were used to calculate adipocyte fatty acid insulin sensitivity (ISI-FFA) by the formula:[2/(insulin × NEFA) +1] [21]. Additionally, pubertal stage was estimated following assessment of fasting plasma concentrations of specific sex hormones (Table 1).

*Dietary variables *included calculation of nutrient intakes and servings of foods consumed by children, following data analysis from 3-day food diary records (Table 1). Additionally, assessments were made of child food habits, preferences, self efficacy and nutrition knowledge; and of family food habits and stages of change with respect to specific healthy food habits.

*Physical activity *of child participants was assessed from 3-day physical activity diary records, and physical fitness was assessed using the Pacer lap run test that is administered as part of the California Fitnessgram evaluations (Table 1). Additionally, child physical activity habits, knowledge, competence and self efficacy were assessed using subjective questionnaires.

*Psychosocial variables *were assessed using subjective questionnaires administered to the child and/or parent (Table 1). These variables included measures of self esteem, body satisfaction, behavior and communications.

*Sociodemographic factors *were measured using subjective questionnaires administered to an adult family member. Factors of interest included age and gender of adults, education, housing and employment status.

*Other subjective data *were obtained also from questionnaires administered to the adult family member. Factors of interest included family history and intrauterine risk of T2DM, diagnoses of girls for polyovarian syndrome, and selected characteristics of the family environment.

### Theoretical model

**Figure 1:**
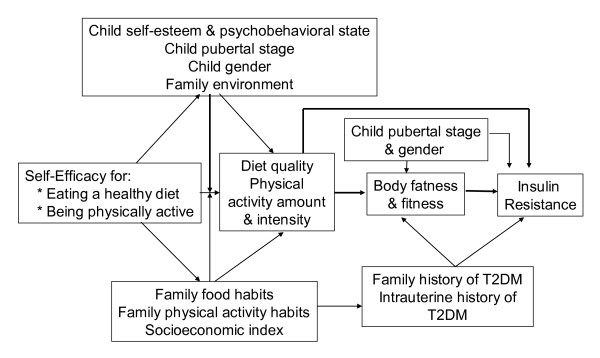
**Theoretical model for *Taking Action Together *study**.

Recognizing the interplay between individual characteristics and one's social environment in determining health-related behaviors, Bandura's Social Cognitive Theory (SCT) was selected as the foundation for *TAT *[22]. In youth, modest improvements in social, cognitive, dietary and exercise outcomes have been achieved when programs utilized SCT [23,24]. Consistent with SCT, we postulated that strong self efficacy would positively affect diet quality, physical activity and energy balance of children which, in turn, would reduce insulin resistance directly or indirectly via effects on body fatness (Figure 1). An extensive research literature has demonstrated that several factors (e.g., child pubertal stage, self esteem, psycho-behavioral status) are able to interfere with or promote these relationships.

### Intervention goals

The development of the *TAT *intervention was informed by a comprehensive review of the literature in conjunction with recommendations from a panel of national experts on policy and program initiatives to prevent T2DM in children [25]. The review revealed that the most efficacious programs aimed to improve both diet and physical activity.

#### Dietary goals

Based on a 2003 review of the dietary determinants of obesity [26], the following dietary goals were selected for *TAT*: increase intake of fruit, vegetables, whole grains, and low-fat dairy foods; reduce intake of high-fat and high-sugar foods and beverages; and sharpen awareness of cues to satiety.

#### Physical activity goals

It has been recommended that children participate in a cumulative total of 60 minutes or more daily of moderate-to-vigorous physical activity [27]. To promote sustained adoption of an active lifestyle, activities should be both fun and doable by children; and overweight children should begin physical activities at a low intensity and duration, gradually increasing as competence is gained [28]. The *TAT *physical activity goal was to increase time spent in moderate-to-vigorous intensity physical activity by developing skills, knowledge and self efficacy with respect to having endurance and being flexible, strong, coordinated, quick and agile.

#### Self-esteem goals

Studies have shown that overweight children tend to have lower body satisfaction and, in some cases, lower self esteem than normal weight children [29]. Exposure to teasing exacerbates the effect [30]. Poor self esteem is often associated with negative behavior [29]. Further, emphasis was placed on self-esteem building since poor self esteem can interfere with the development and maintenance of positive eating and physical activity behaviors, and since efforts to improve these behaviors can unintentionally negatively influence self esteem. Our goal for the self-esteem component was to develop five social and emotional competencies:awareness of one's strengths, challenges, behaviors and styles of interacting; ability to effectively interact and communicate with others; respect for self and others; ability to describe self using art, writing and verbal means; and ability to assess positive change in one's behavior and personal interactions. Consistent with the tenet of 'health at every size' [31], goals for body weight were not instituted. Improving insulin sensitivity, regardless of weight loss, has been reported to reduce risk of metabolic disease [32]. Further, to avoid stigmatization due to body fatness, focus was placed on improving diet and physical activity rather than on body weight.

#### Consultation with multi-disciplinary and community experts

To inform the multiple components of *TAT*, we collaborated with a team of experts specializing in a wide range of disciplines including nutrition, exercise physiology, public health, psychology, medicine, education, and cultural sensitivity (Table 2). In addition, advice was sought on curriculum, program activities, participant retention strategies, opportunities for program enhancement and expansion, and venues for disseminating research findings.

**Table 2:** Coordinated and Complementary Roles of Research Staff, Consultants and Advisory Board Members for the *Taking Action Together *Project

EXPERTISE	UCB Researchers	Collaborators & Consultants^1^	Board members^2^
**INTERVENTION**			
Adult education	√		√
Cultural relevance		√	√
Curriculum	√		√
Family involvement		√	√
Medical management		√	
Nutrition	√		√
Physical education	√	√	√
Process evaluation	√		
Self-esteem	√	√	√
Study management	√		

**EVALUATION**			
Anthropometry	√	√	√
Biochemical	√	√	√
Diet	√		√
Family demographics & characteristics	√	√	
Physical activity & fitness	√	√	√
Self-esteem & behavior	√	√	√
Statistics & data analysis	√		

**DISSEMINATION**			
Behavioral scientists		√	
Cooperative Extension	√		
Medical community	√	√	√
Research scientists	√	√	√
YMCA and other after-school/community venues			√

^**1 **^Academic collaborators and consultants included researchers at University of California (UC) Berkeley, UC San Francisco, UC Riverside, Brock University in Toronto, and UC Cooperative Extension.

^**2 **^Advisory board members included representation from diverse programs including the Food Stamp and Nutrition Education Program (youth and adult programs), Expanded Food and Nutrition Education Program (EFNEP), 5-a-Day Power Play, Sports Play and Active Recreation for Kids (SPARK), California Adolescent Nutrition and Fitness Program (CanFIT), and the YMCA of the East Bay. Other collaborating institutions and organizations with a long history of involvement in the inner-city Oakland community included Children's Hospital and Research Institute Oakland, the California Department of Health Services and UC county-based Cooperative Extension.

The *TAT *Advisory Board (Table 2) included community members who had successfully developed relevant programs for school-aged children and their families; and members with experience working specifically with inner-city residents and low-income African American families. The Advisory Board was convened yearly; ad hoc consultations with individual members were undertaken as needed. Additionally, we capitalized on established and essential working relationships with local community and state partner organizations including county-based Cooperative Extension programs, and federal USDA-funded food supplementation programs such as FSNEP and EFNEP.

### Control group programming

In contrast to the intervention group, contact with child participants and families in the control group occurred primarily during recruitment and yearly data collection, and there was no hands-on involvement in intervention activities. To encourage participation and maximize study retention while minimizing the 'Hawthorne' effect [33] and dissatisfaction with minimal intervention, control families received information focusing on community opportunities for health promoting activities in the mail monthly, and control children were offered a free week of traditional YMCA summer day camp.

### Trial design, participant recruitment and group assignment

#### Trial design

This controlled, prospective, non-randomized trial was approved by the institutional review board (ethics committees) at the University of California -- Berkeley and San Francisco. Parental informed consent was obtained for all participants. To achieve adequate sample size, three cohorts of participants were recruited over three successive years (Table 3).

**Table 3:** *Taking Action Together *Project Plan - Participants by Group

	Control group	Intervention group
Baseline	Number of participants (boys; girls)	
Cohort 1	51 (21; 30)	52 (20; 32)
Cohort 2	28 (16; 12)	35 (16; 19)
Cohort 3	32 (14; 18)	37 (16; 21)
Total	111 (51; 60)	124 (52; 72)

End of 1^st ^Year Follow-up		
Cohort 1	33 (13; 20)	37 (14; 23)
Cohort 2	20 (11; 9)	18 (7; 11)
Cohort 3	14 (9; 5)	14 (7; 7)
Total	67 (33; 34)	69 (28; 41)

End of 2^nd ^Year Follow-up		
Cohort 1	26 (10; 16)	24 (10; 14)
Cohort 2	12 (6; 6)	11 (7; 4)
Total	38 (16; 22)	35 (17:28)

#### Recruitment

Announcements were distributed at elementary schools and other venues within the targeted communities. Incentives, benefits and risks to participants were conveyed in these announcements. Recruitment was restricted to community venues in regions of Oakland habitated by low income African American families. Because families in these regions tend to be fluid, no restrictions were placed on family structure, ethnicity of the adult caregivers or relationship to the child participant. Data were collected from only 1 child per family. As it happened, similar numbers of families self-selected into each of the two sites and similar numbers were included at baseline (Control group, n = 111; Treatment group, n = 124; Table 3). We expected to achieve a 50:50 ratio for boys:girls, and achieved a ratio at baseline of 44:56.

#### Assessing bias due to motivation

The potential for bias due to motivation was estimated by comparing dropout rates between the two groups during the 1 or 2 year intervention period. Dropout rates for control and intervention groups were similar after both 1 year (40% versus 44%; p = 0.46) and 2 years (66% versus 72%; p = 0.52) of programming. Furthermore, when information collected at baseline was compared for dropouts from the control versus treatment groups, no significant differences were found. As examples, mean baseline insulin resistance (HOMA-IR) values of children who dropped out of the control versus treatment groups during the first year were 2.61 and 2.76, respectively (p = 0.67), and means for dropouts during two years of programming were 2.59 and 2.55, respectively (p = 0.91). Additionally, baseline insulin resistance values for children who completed two years of programming versus those who did not were not significantly different (HOMA-IR mean values: 2.56 and 2.53, respectively; p = 0.90).

#### Reducing bias due to non-randomization

**Figure 2:**
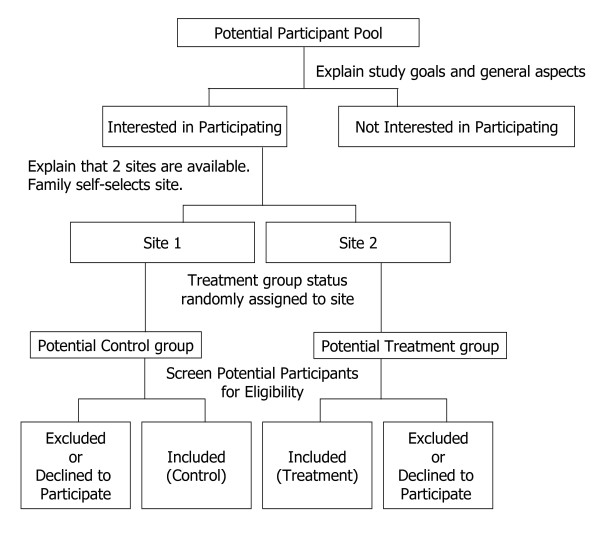
**Flowchart of study participant recruitment, self-selection to site, and eligibility assessment**.

Families self-selected to attend programming at one of the two sites, and this selection preceded information regarding the assignment of treatment to that site; ie. when selecting a site, families did not know whether they would be in the control or the intervention group (Figure 2). Prior to beginning Wave 1, and after families selected their preferred site, a toss of the coin was used to determine which of the two sites would be the intervention site. For subsequent waves, families still were unaware of treatment status when choosing their preferred site. The two sites were similar in many respects including their staffing, characteristics of the facility, economic and cultural characteristics of neighborhood residents, crime rates, and general lack of health care, supermarkets and parks. Although contamination across sites may not have been entirely eliminated, staff (research and YMCA) were alerted to report any interactions that could suggest contamination. We were not made aware of any such reports during the 3-year intervention period.

At the analytical stage, statistical analyses for differences by treatment group will be controlled, if needed, for covariates including child age, gender, socioeconomic status, pubertal stage or other potential anthropometric, dietary, physical activity, self esteem, behavior or family confounders (for a comprehensive list of variable assessed, refer to Table 1). The necessity for adjustment for a particular variable will be determined in a preliminary analysis in the following way. Any variable modestly related (p < 0.25) to BOTH treatment group status and the change in the outcome variable will be considered to be a potential confounder and included in analytic models. Since subjects were not randomized into groups, analyses will be statistically adjusted for baseline outcome values (analysis of covariance) as groups may not have been equivalent at baseline for these outcomes (some examples are shown in Table 4).

**Table 4:** Some baseline characteristics of participants in the control and intervention groups (p-value for difference by group).

	Characteristic	Control (mean)	Intervention (mean)	p-value
Child	Age (yr)	9.84	9.85	0.92
	BMI z-score	2.00	2.05	0.52
	Waist-to-hip ratio	0.88	0.89	0.36
	% body fat	36.2	36.3	0.90
	Pubertal stage (1-5 scale)	2.28	2.17	0.54
	Fasting HbA1c (%)	5.18	5.14	0.44
	Fasting HOMA-IR	2.49	2.51	0.94
	Fasting ISI-FFA	0.43	0.42	0.72
	Energy intake (kcal/day)	1829	1887	0.53
	Fat intake (g/day)	75.2	73.0	0.62
	Carbohydrate intake (g/day)	220	245	0.04
	Protein intake (g/day)	71.5	67.9	0.38
	Whole grains intake (servings/day)	0.70	0.73	0.77
	Fruit & vegetable intake (servings/day)	2.06	2.38	0.10
	Dairy intake (servings/day)	1.27	1.21	0.59
	Nutrition knowledge (1-22 scale)	9.63	9.96	0.25
	Food preferences (7-25 scale)	16.0	16.3	0.32
	Snack habits (1-5 scale)	2.41	2.45	0.76
	Fitness (ml O2/min/kg body wt)	41.8	43.8	0.26
	Moderate- & high-intensity physical activity (min/day)	89.7	104.4	0.31
	Global self-worth (1-4 scale)	2.98	3.04	0.55
	Athletic competence (1-4 scale)	2.76	2.82	0.47
Family or Adult	Age of adult responder (yr)	37.1	38.5	0.30
	SEI (4-12 range)	8.42	8.29	0.59
	Family History of T2DM (0-2 scale)	0.13	0.20	0.18
	Intrauterine risk for T2DM (0-2 scale)	0.34	0.32	0.82
	Food habits (7-36 scale)	21.9	23.0	0.04
	Physical activity habits (3-15 scale)	11.7	12.2	0.28
	Family Cohesion (1-4 scale)	3.47	3.45	0.81
	Family Conflict (1-4 scale)	0.94	0.85	0.38

### Data management and statistical analyses

The project was designed to allow change in outcome variables to be calculated for each participant using a pre-post experimental design. Measures were taken at baseline prior to exposure to intervention, then again following either 1 or 2 years of programming (Table 5). To achieve a balanced design, similar numbers of control and treatment participants were recruited into each of the three cohorts, and similar numbers of boys and girls were recruited into each treatment group (Table 3). Data for cohorts will be combined at the analysis stage.

**Table 5:** *Taking Action Together *Project Plan - Project Timeline

Project Year^1^	**1**^3^				**2**^3^				**3**^3^				**4**^3^			
Develop program	A	B	C	D												
Refine program					A	B	C	D	A	B	C	D				
Convene Advisory Board		B				B				B				B		
Train staff			C	D	A	B	C	D	A	B	C	D	A	B	C	
Conduct process evaluation					A	B	C	D	A	B	C	D	A	B	C	D

Recruit study participants																
First cohort (n = 103)			C	D												
Second cohort (n = 63)							C	D								
Third cohort (n = 69)											C	D				

Provide intervention^2^																
First cohort					A	B	C	D	A	B	C	D	A			
Second cohort									A	B	C	D	A	B	C	D
Third cohort													A	B	C	D

Perform impact measures (0 = pre-study;1 = 1^st ^Yr follow-up; 2 = 2^nd ^Yr follow-up)^2^																
First cohort				0				1				2				
Second cohort								0				1				2
Third cohort												0				1

^1 ^Project began middle of 2004 and ended in middle of 2008.

^2 ^Quartiles of each study year are indicated as follows:  A, 1^st^ quartile; B, 2^nd^ quartile, C, 3^rd^ quartile, D, 4^th^ quartile.

^3^ The first and second cohorts were followed for a total of two years whereas the third cohort was followed only for a single year.

#### Quantitative data

Quantitative data were managed using computer programs including Microsoft Excel, EpiData, BASC-2 software [34], USDA Nutrient Database [35] and MyPyramid Equivalents Database [36]. Data were double entered and edit checks were incorporated into the data entry program to identify data entry errors. For psychosocial, knowledge and attitude measures, scales will be developed as necessary and compared as described above.

Mean changes in the quantitative outcome variables of interest (HOMA-IR and secondary anthropometric, dietary and activity variables) over one or two intervention years will be evaluated using multiple regression techniques. Adjustment for multiple comparisons will not be made, and with values for p < 0.05 considered statistically significant [37]. In addition, because some of these outcome measures are considered to be intermediary or confounding variables, we will use multiple linear regression to examine the effect of intervention on the primary outcome variable of interest (HOMA-IR), adjusting for baseline HOMA-IR, BMI z-score, psychosocial measures such as self esteem, diet and physical activity behaviors, and other potentially relevant factors such as family and (to account for genetic differences in response to intervention) intrauterine history of T2DM. The correlations of all potential covariates with both the dependent and independent variables will be examined, and variables that correlate at p < 0.25 with both variables will be included in the models.

Initially, we will examine the regression coefficient for intervention status (a dummy variable where 1 = intervention group, 0 = control group) in a simple regression model. Using change in HOMA-IR as one dependent variable example, we will model as follows:

Next, we will examine the effects of other explanatory and/or confounding variables to determine whether they explain the effects of intervention status (if any) using as an example:

#### Qualitative data

Qualitative data from interviews with families will be transcribed and analyzed using grounded theory, a qualitative approach that is used to analyze social processes present within human interactions [38]. Application of the theory can result in explanations of important family processes or structures that are grounded in the empirical data. Major themes can be inductively derived from the data to articulate the dynamics of psychosocial family environment.

### Statistical power and sample size

When this study was originally conceptualized in 2003, few data were available in the literature describing the variability in insulin resistance, assessed using fasting HOMA-IR, among overweight African American children. Thus, since it was expected that the qualifying children would be hyperinsulinemic but not hyperglycemic, sample size was estimated using values for plasma insulin. For evaluation of the effectiveness of the intervention, a sample of 50 children in the intervention group and another 50 in the control group was determined to allow detection of a difference in plasma insulin levels of 4 μU/ml, assuming an initial fasting plasma insulin of 30 μU/ml [39-41], a standard deviation of 7.2 μU/ml [42] and a correlation coefficient of 0.6 between initial and final values in the control group (Type I error = 0.05, and Type II error = 0.20). A total sample size of 150 participants at baseline (75 per group) was initially planned for, based on an estimated yearly attrition rate of 33%. Because the attrition rate was higher than anticipated following the first year of Wave 1, the number of participants was increased during the course of this study.

### Training

#### Control staff

YMCA staff at the control site received the in-house YMCA training provided to all new staff, and a limited 1-hr orientation to the project.

#### Intervention staff

Implementers of the TAT intervention included UC Berkeley staff and students, and YMCA staff. The adult-to-child ratio was 1:8 for most intervention activities. Site instructors and key YMCA staff received in-house YMCA training, plus program-specific training prior to commencement of each program year, followed by weekly and monthly training (referred to hereafter as "initial" and "continuing" training, respectively). Initial training aimed to provide an understanding of expectations with respect to participant confidentiality, safety and communications; the nature of each curriculum component; and the influence of self esteem and of eating and physical activity behaviors on risk of T2DM. Continuing training focused on team building (3 hr/yr), health lifestyle challenges in an obesigenic environment (3 hr/yr), managing challenging behaviors in children (8 hr/yr), use of social cognitive theory in practice (2 hr/yr), nutrition lesson-specific training (40 hr/yr), physical activity lesson-specific training (10 hr/yr), self-esteem lesson-specific training (10 hr/yr), and strategies for motivating families (Table 6).

**Table 6:** Training Components and Content

Program Component	Training time	Content
1. Introductions, team-building, project overview^1^	1 hr during initial and yearly training (+3 hr additional during year-long program)	Introduce personnel roles. Describe the research project and program. Build team atmosphere. Introduce Body Positive and Health at Every Size concepts.
2. Healthy lifestyle challenges in an obesogenic environment^2^	1 hr (+0 hr)	Introduce nutrition, physical activity and self-esteem goals. Discuss challenges African American families experience in inner-city Oakland in efforts to live a healthy lifestyle.
3. Managing challenging behaviors in children^2^	1 hr (+8 hr)	Introduce positive behavioral management approaches and describe how to effectively develop social skills and problem-solving abilities in children.
4. Social cognitive theory (SCT) in practice^2^	1 hr (+2 hr)	Understanding and using self-observation, peer modeling, feedback, verbal encouragement, mastery through trials of increasing difficulty.
5. Nutrition^2^ General Lesson-by-lesson	1 hr (+2 hr) 0 hr (+40 hr)	Describe components of healthy diet, considerations of African American culture, income, food availability and transportation; demonstrate use of SCT3, importance of fun.
6. Physical activity^2^ General Lesson-by-lesson	1 hr (+2 hr) 0 hr (+10 hr)	Discuss barriers, use of Health at Every Size concept and SCT to increase activity self-efficacy, motivational strategies, cultural preferences and considerations.
7. Self-esteem^2^ General Lesson-by-lesson	1 hr (+2 hr) 0 hr (+10 hr)	Describe contributors to and effect of positive self-esteem, cultural differences in expression. Discuss strategies to develop self-esteem using Body Positive approach, preventing harm.
8. Motivating families^2^	1 hr (+1 hr)	Influence of parenting style, family food and activity practices, and community environment on development of healthy behaviors; motivational strategies; cultural considerations.

^1 ^Separate sessions for Control and Intervention site staff.

^2 ^Intervention site staff only.

### Intervention curricula and delivery to children

Each program year began with a kick off to integrate all elements of the curricula in a fun-filled, action-packed 2 weeks of YMCA day-camp. Children were provided ample opportunities to bond with each other and staff to promote continued participation. A celebratory family event was held at the end of the 2 weeks for children to showcase the program to their families.

Yearly schedule of child activities included 10 days (8 hr per day) of program-specific summer day-camp, plus 1 (2-hr) session/wk, 3 weeks per month, 11.5 month for a total of 150 hr (50% devoted to physical activity and 25% devoted each to nutrition and self-esteem building) for children.

With goals and a theoretical model for *TAT *as a guide, we examined relevant curricula from peer-reviewed scientific literature and on-line sources. Materials were evaluated for: age-appropriateness; socio-cultural relevance; body weight neutral (incorporating a 'health at every size' approach); current within 10 years; experiential (a hands-on, rather than didactic, approach to learning); and resonance with program goals. No single program curriculum was identified that met all project criteria. Thus, select materials were used from a variety of curricula and, as needed, adapted for use with our target population.

#### Educational components

Lessons and activities focused on improvements in dietary quality and quantity, increased time in moderate-to-vigorous activity, and healthy self-esteem building through strengthening of cultural pride and social and behavioral competence. Conceptual elements included knowledge and skill building, modeling, goal setting, self-assessment, practice and reinforcement. Educational strategies included individual practice, cooperative learning, and individual and group discussions. Modules (6 modules, 5 weeks each), and lessons within modules, were sequenced to equip children with knowledge, skills, self efficacy and intentions to make healthy diet and physical activity choices. Examples of lessons delivered in this program are available online http://www.cnr.berkeley.edu/cwh/.

#### Nutrition component

The core nutrition lessons utilized the approach of "The Power of Choice: Helping Youth Make Healthy Eating and Fitness Decisions" [43], and included 'hands-on" learning via preparation of low-cost, culturally appropriate foods, taste testing, and exposure to new foods and ingredients. Lessons were conducted with a minimum of equipment in modest facilities, and aimed to develop knowledge, challenge children to identify healthy options and make healthier food choices within usual settings of fast food outlets and corner stores, set personal goals, and self-assess in order to refine goals. Nutrition topics were sequenced as follows: making low fat choices, increasing fruit and vegetables, replacing sugar and portion size, replacing refined with whole grains, healthy snacks and hunger cues, moderation and a balanced diet.

#### Self-esteem component

Core self-esteem lessons were developed based on the widely-used curricula, Body Positive [44], a program designed to promote health at every size, body satisfaction and self-esteem, and Kwanza-based activities designed to promote cultural pride and build community. As needs became evident, health and behavior lessons were adapted from the approach of Kids' Health [45] to address topics such as hygiene, acne, puberty, anger management, bullying, and being teased. Self-esteem topics were sequenced as follows: self awareness & cultural expression of self, positive interaction, effective communication, Body Positive, 'Health At Every Size' & respect of self and others, taking charge of your personal hygiene, overcoming challenges (including bullying, teasing and conflict), self-assessment of personal challenges, and positive growth.

#### Physical activity component

Activities were designed to consider the body weight and fitness status of children, with gradual and manageable increases in difficulty, duration and frequency. Activities, based on the After-School SPARK [46] physical activity program, were child-centered, encouraged enthusiasm and participation, and aimed to increase enjoyment through games, dance and sports. Physical activity programming was sequenced as follows: flexibility, strengthening, endurance, balance and coordination, speed and agility. Children were encouraged to be active daily even when family members or peers were not, including outside of *TAT *program time; for this purpose all children received a free YMCA membership, allowing access to the facility. Although we acquired evidence that children used these free memberships to utilize the YMCA facilities, their effectiveness at increasing the overall activity of children outside of program could not be selectively determined within the design of this study.

### Parent programming

Studies of obesity prevention have shown that parental involvement is critical to substantively alter children's dietary intakes and energy expenditures [47]. For adult family members, monthly mailing of health education materials, educational meetings (including advertisements about other free, family-focused healthy lifestyle events available in the community that supported TAT goals), phone calls, and/or in-home visits to target overcoming barriers to adopting healthy behaviors, were scheduled. Also 3 healthy lifestyle events and family celebrations were scheduled for intervention group children and families. At the completion of year-end evaluations and data collection, a celebratory family event honored and acknowledged the gains of the child participants.

Parents/guardians were invited also to nine 1-2 hr sessions each year. The curriculum for adult family members in the intervention group relied on the interactive (discussion, information, and food & physical activity demonstrations) "Eating Smart. Being Active" adult EFNEP lessons regarding the importance of physical activity; meal planning, food shopping, reading food labels; increasing intakes of vegetables, fruit, whole grains and calcium-rich foods; portion control; and approaches to limiting intakes of fat, sugar and salt http://efnep.ucdavis.edu/AdultCurriculum.html.

### Outcome and process evaluation

Data collection (Table 1) was designed to allow the effects of intervention to be determined on the primary outcome variable (insulin resistance) and on intermediate outcomes (dietary intakes, physical activity). Changes in behaviors (self efficacy, self esteem) expected to precede changes in primary and intermediate outcomes also were assessed. Finally, collection of data for potential modifiers facilitated increased statistical power by their inclusion, if indicated, as covariates. Outcome evaluations were performed at baseline, and after one and two years of intervention. To maximize objectivity, evaluation staff were different from intervention staff. Process evaluation data were collected to assess the quantity and quality of program services delivered, and responses of child participants, adult participants, paid staff and volunteers (Table 7).

**Table 7:** Process Evaluation Data Collection in *Taking Action Together*

Variables	Methods and Frequency^1^
Adherence to schedule of lessons	Observation - monthly for child component; twice per year for adult component Log - recorded weekly; reviewed monthly
Fidelity of lesson delivery	Observation - monthly for child component; twice yearly for adult component Session evaluation forms - recorded weekly, reviewed monthly
Training, support and monitoring of staff	Observation of attendance and engagement Observation of lesson delivery - bimonthly (child component), twice per year (adult)
Response of participants to sessions	Attendance log - by session; reviewed monthly Session evaluation form - administered lesson-by-lesson (child component), twice per year (adult)
Response of staff and volunteers to sessions	Session evaluation form - administered lesson-by-lesson for child sessions Meeting of staff and volunteers - twice per year
Family response to mailings	Telephone survey - contact intervention families once per year
Overall assessment of program	Confidential telephone survey of intervention families - end of year

## Discussion

### Lessons learned

#### Glucoregulation

Analyses performed to date show that the intervention program stabilized or improved glucoregulation in children after 1 year, the effect being larger in boys than in girls [48]. Also, a larger percentage of children in the treatment group (vs. control) decreased BMI z-scores after 1 yr.

#### Additional behavioral components for children

Before adequate focus could be placed on health topics, we learned that children needed to develop tools to modify their own behavior. As needs became evident, additional curricula components were developed to address these needs. Furthermore, to manage behavior, create a better learning environment, and increase enjoyment, staff was trained to use positive behavioral modification strategies in all program components. To meet these under-anticipated needs, additional staff training was incorporated, and on-going training and staff support was provided by mentoring and weekly discussion. Further, lessons were developed as needed to address community (eg. neighborhood violence), national (eg. Hurricane Katrina) or international (eg. flooding) disasters that affected the participants and their families. We have recently reported that parent-reported post-intervention scores favored the intervention condition for three of the four key psychobehavioral composities that were evaluated cross-sectionally [49].

#### Additional strategies to engage parents

Attendance of adult family members at family-focused programming was low due to numerous compelling and competing demands (single parent households, multiple jobs, other children). To address this and facilitate on-going communication, three alternative forms of engagement were implemented -- telephone calls, home visits, and monthly newsletters. Telephone calls were provided weekly or monthly as needed to help families address barriers to targeted behavioral change and promote family support of child participants. Study staff also provided this support during in-home visits. Families responded very favorably to telephone communication and in-home visits, particularly during times of family struggle, citing the "bridging" effect this created between family and program, an effect that increased program retention. Monthly newsletters were provided which included recipes the children made, monthly programmatic goals, information on free and low-cost health-promoting community activities, and educational materials regarding the importance of healthy food choices and physical activity.

#### Transportation issues

Even though children were recruited from neighborhoods near the YMCA sites, we learned early that attendance was improved if rides were provided for after-school and evening events. Transportation played an important role, as children were not allowed by their families to walk or bicycle to the facilities because of safety concerns. While this was an unforeseen cost (necessitating the hiring of a van and van driver), it improved attendance and reliability of outcome measures for this proof-of-concept pilot project. Subsequent implementation efforts should avoid this issue by delivering the program within established after-school settings.

#### Community bonds

Participation in this study proved to be an intense and influential experience for all involved. Staff gained new respect for the resourcefulness of children and their families, and were struck by the immense negative effect of neighborhood violence on attitudes, behaviors and activities. Family members, who were initially reserved and distrustful, later expressed gratitude that their children could be truly supported and cared for by persons outside their immediate community. Although we initially focused on the importance of a sound educational strategy, we later placed this third - after providing for the primary needs of participants, and the behavioral skills needed to receive the programming offered to them. Lastly, while we initially provided inexpensive incentives for attendance (e.g., athletic equipment, recipe books), these failed to serve their intended purpose. Instead, the children and families responded most profoundly to involvement at the personal level. Subsequent implementation efforts should seek strong interpersonal skills in staff and volunteers.

### Interpretation

We attempted to incorporate into *TAT *the lessons learned from previous programs to prevent T2DM in children. Specifically, we included a multi-disciplinary team, attempted to involve parents/guardians, targeted the most promising nutrition and physical activity goals for intervention, emphasized experiential learning, and developed and delivered a program that children enjoyed and wanted to attend.

Although nutrition and physical activity have been the usual targets of T2DM prevention programs, the inclusion of self esteem is unique to *TAT*. Additionally, *TAT *included a psycho-behavioral component that was essential to address the unique challenges faced by low-income, inner-city children. To our knowledge this is one of few studies to evaluate both biological and sociological responses to an intervention in children. Unlike school-based programs, this non-school program has the potential to be a sustainable, highly accessible physician-referral program for children identified at high risk of weight gain and associated complications.

Several opportunities were noted for program expansion by inclusion of other ethnic groups, and intensification via daily delivery in after-school venues. The low-income, inner-city African American population targeted by *TAT *faces a higher risk of chronic disease compared to other groups, a gap that continues to widen [5], and a disproportionate share of obstacles -- single parenthood, job insecurity, violence, inadequate medical care, discrimination, and suboptimal housing. An intervention that can successfully overcome *these *barriers may, with comparative ease, be translated to other populations. Demonstrating efficacy in other ethnic groups will suggest generalizability of the intervention for inner-city American youth.

It is unrealistic to expect healthy behaviors to become ingrained after one or even two years. However, demonstrating significant impact in one year is justification for continuation. Our long-term goal is that *TAT*, with minimal external support, could be institutionalized and continued long after the research is over, extending reach to many more children and families in other out-of-school settings here and elsewhere.

In conclusion, this protocol evaluates the theory of self efficacy to improve children's dietary intake, physical activity, and self esteem. Improved glucoregulation in children after this intervention [48] suggests that the TAT protocol has a great potential to serve as a guideline protocol for future interventions in children's health related area; and the more favorable psychobehavioral scores for children in the intervention group [49]suggest potential for improvements in characteristics that may broadly influence a child's ability to be successful in school and elsewhere. Assessment of the extent to which the program achieves its aims will depend on approaches, designs and other individual factors related to the area of intervention.

### Limitations

This pre-post non-randomized experimental design did not allow differences due to program site to be evaluated, since the control program was delivered at one site and the treatment program at another. A follow-up site-randomized controlled study would be needed to determine whether similar differences between treatment group status would be observed under this more rigorous design. Results suggest that such a follow-up study is justified. This study also did not evaluate efficacy is children either younger than 9 yr or older than 10 yr; it did not evaluate efficacy in children of ethnicities other than African-American; and it did not evaluate efficacy in middle-or upper-income populations. Until additional studies are performed, these data cannot be extrapolated to the breadth of children in America or elsewhere.

## Competing interests

The authors declare that they have no competing interests.

## Authors' contributions

**LDR **was involved in conceptualizing the study, implementation, evaluation and manuscript preparation. **SS **was involved in manuscript preparation and submission. **JI **contributed in conceptualizing the study. **RAM, AR **and **BSG **were involved in implementation and evaluation. **SEF **supervised conceptualizing of the study, implementation, evaluation and manuscript preparation and was also the Principle Investigator of the intervention.
